# Waste Eggshells as a Natural Filler for the Poly(Vinyl Chloride) Composites

**DOI:** 10.3390/polym14204372

**Published:** 2022-10-17

**Authors:** Katarzyna Skórczewska, Krzysztof Lewandowski, Piotr Szewczykowski, Sławomir Wilczewski, Joanna Szulc, Paulina Stopa, Paulina Nowakowska

**Affiliations:** 1 Department of Polymer Technology and Protective Coatings, Faculty of Chemical Technology and Engineering, Bydgoszcz University of Science and Technology, Seminaryjna 3, 85-326 Bydgoszcz, Poland; 2 Department of Manufacturing Techniques, Faculty of Mechanical Engineering, Bydgoszcz University of Science and Technology, Kaliskiego 7, 85-796 Bydgoszcz, Poland

**Keywords:** eggshell waste, poly(vinyl chloride) (PVC), PVC composite, structure, properties, scanning electron microscopy analysis, micro-CT analysis

## Abstract

The paper presents the characteristics of unplasticized PVC composites modified with biofiller obtained from the waste eggshells of hen eggs. The composites obtained by extrusion contained from 10 phr to 40 phr of biofiller. The filler was characterized using the SEM, TG, and sieve analysis methods. The influence of the filler on the processing properties was determined using plastographometric and MFR tests. Fundamental analysis of mechanical properties was also performed, i.e., Charpy impact strength and determination of tensile properties. The mechanical properties were supported with dynamical mechanical thermal analysis, time of thermal stability, and thermogravimetric analysis. Structure analysis was also performed using SEM and X-ray microcomputed tomography (micro-CT). The processing properties of the tested composites do not give grounds for disqualifying such material from traditional processing PVC mixtures. Notably, the biofiller significantly improves thermal stability. Ground eggshells (ES) work as scavengers for the Cl radicals released in the first stage, which delays the PVC chain’s decay. Additionally, a significant increase in the value of the modulus of elasticity and softening point (VST) of the composites concerning PVC was found. Ground hen eggshells can be used as an effective filler for PVC composites.

## 1. Introduction

Hen eggshells are food waste from poultry hatcheries and the production of food products (e.g., cakes, pasta, egg products) but also from households. As a byproduct, they are a significant problem for producers both economically and ecologically. Most of the eggshell waste ends up in landfills without further processing.

In 2018, world egg production was about 71 million tons [1], of which China accounted for 35% of world production, North America for 12%, the European Union for 10%, and India for 7%. Considering that 11% of the average weight of an egg is a shell, each year 7.8 million tons of eggshells are produced as waste worldwide. The largest egg-producing countries in Europe are France, Germany, Spain, Italy, and Poland [2,3]. In Poland, almost 11 billion eggs (for consumption and hatching) were produced in 2021 [4]. Eggshell introduces an excellent ecofriendly source of biogenic minerals with opportunities to replace synthetic or mine mineral CaCO_3_, especially in many materials applications.

The main component of the eggshell is calcium carbonate in the form of calcite (94%). Apart from CaCO_3_, there are also other inorganic compounds in the eggshell (ES), e.g., magnesium carbonate (<1%), calcium phosphate (<1%), and silicon oxide (<1%). The remaining approx. 4% of the shell mass consists of polysaccharides, various collagens, fatty acids, and water [5,6]. The eggshell microstructure is unique. The CaCO_3_ skeleton is characterized by a hierarchical three-level porous, rough structure with primary particle sizes of about 10 nm. Calcite crystals are arranged into palisade and mammillary layers with different morphology and porosity [7].

However, more and more often, due to their valuable organic and inorganic components, ways of their reuse are sought. There is growing interest in the different ways eggshells can be used. Waste eggshells are a valuable functional raw material and supplement in the production of baked goods [8,9] or yogurts [10]. This material is also valuable in cosmetics and pharmaceuticals production [11]. They can be a significant raw material for producing nanoparticles used later as absorbers of pollutants [12,13,14], especially heavy metals [15]. They are also used as a substitute material in the production of cement or aggregate [16,17]. Other sources indicate their use as soil conditioners, fertilizers, and absorbents for removing dyes and heavy metals [18]. Powdered shells are a source of calcium carbonate, which, after appropriate preparation, can be used as a dietary supplement, cosmetic substrate, or as a feed additive. Chicken eggshells are biodegradable and biocompatible. As a result, they used to produce new biomedical materials. It is possible to obtain fillers for bones and implants or biologically neutral implants when used [19]. Crusts can also be a valuable hydroxyapatite source, especially in biomedical applications [20].

The biofiller obtained from eggshells as a source of calcium carbonate could be successfully used to modify polymer materials. The eggshell’s fascinating and developed nanoporous structure can lead to a complex interaction of the ES with the polymer matrix. More and more scientific reports show the results of using biogenic egg filler to modify polymers based on polystyrene, polypropylene, polypropylene, epoxy, NBR, LLDPE, HDPE, PLA, PVA, and PLA [21,22,23,24,25,26,27]. The ES in epoxy resin has functioned as a flame retardant and smoke suppression modifier [28]. These features were also tested in the case of PLA [29]. Additionally, a number of treatments of this waste are used, for example, in calcination [30], phosphorus decoration [31], or the adsorption of metallic species [32].

Poly (vinyl chloride) is one of the most processed thermoplastics. In industrial practice, this polymer is often subject to the modification of its properties, e.g., by using plasticizers, process agents, and various fillers, including waste fillers [33,34]. It is essential to obtain materials with improved thermal stability [35,36]. A commonly used additive in PVC-based plastics is calcium carbonate. Its share in the material can be over 50%. It is introduced to reduce polymer consumption, which is associated with cost reduction. It also increases the modulus of elasticity and hardness and improves the quality of the product’s surface. Such composites are used, for example, in the production of window and furniture profiles.

Despite the growing interest in using ES as a filler for polymers, there is little information on using eggshell waste for PVC modification. The influence of eggshell particle size and the method of ES introduction into the mixtures on the properties of plasticized PVC were analyzed in the works [37,38]. It was found, among other things, that particles with a smaller size more effectively improve the mechanical properties, which is related to better filler dispersion. On the other hand, higher thermal stability values were noted when using larger size ES particles for modification. ES, together with ZnO, was also used to prepare the PVC heat stabilizer, the effectiveness of which was confirmed on samples obtained with the solvent method [39]. Chicken eggshells were also used to produce a plasticizing–stabilizing modifier by synthesizing calcium soaps from epoxidized vegetable oils [40]. Powdered ES was also used as a bioactive coating, among other things, for PVC coating by the plasma spray coatings method. Obtaining a hard layer on the PVC substrate enhances the storage modulus [41].

The apparent research issue is managing the growing waste stream from the food and poultry industry. Due to the different nanostructure of eggshells, it is likely to produce material with other properties compared to traditional chalk filler. Taking the above into account, it seems an exciting direction to conduct work to assess the possibility of using waste chicken eggshells as a filler for composites based on unplasticized PVC. An additional premise that may be noted in the literature is that chicken eggshells favor more effective dechlorination of PVC waste during their thermal recycling [37,42,43], which, in the perspective of the sustainability and monitoring of the product life cycle, is an added value.

The study aimed to determine the possibility of managing waste such as chicken shells as a biofiller for the production of composites based on unplasticized poly(vinyl chloride).

## 2. Materials and Methods

### 2.1. Materials

A PVC dry blend was used as the matrix of the composites. The PVC dry blend formulation was composed of: suspension PVC type Neralit S-601 (supplied by Anwil S. A Orlen Group Włocławek, Poland)—100 phr; organotin stabilizer Patstab 2310 (Patcham, Netherlands)—2 phr; Lubricant Ceasit (Baerlocher Production, Cincinnati, OH, USA)—1.2 phr; the paraffin wax Naftolube FTP—0.5 phr (Chemson, Austria); Loxiol G-32—1.5 phr; Paraloid K-125—1 phr; Paraloid K-175—1 phr.

Waste in the form of scruples was used to produce the filler, such as domestic “Rosa” breeds produced on the farm (Mała Cerkwica, Poland).

### 2.2. Eggshell Filler (ES) Preparation

To separate the organic residues of the protein membrane, the obtained waste in the form of mechanically crushed eggshells was soaked in water for 24 h and then washed several times until the secretion of these membranes ceased. The shells obtained without organic fraction were dried to a constant weight at 80 °C and ground with a ZM200 Retsch ultracentrifugal mill (Haan, Germany). The resulting ground material was sieved with a vibratory sieve shaker ANALYSETTE 3 PRO, Fritsch (Idar-Oberstein, Germany) to remove the 125 μm mesh size remaining on the sieve. The material prepared in this way was a filler for PVC modification.

### 2.3. PVC/ES Composites Processing

A weighed amount of ES was introduced into the previously prepared PVC dry blends according to the composition given in the description of the materials and mixed with a high-speed mechanical stirrer, Ika Eurostar 6000, for 5 min. The homogeneous mixture was processed by extrusion with a laboratory single-screw extruder (Brabender GmbH & Co., Duisburg, Germany) equipped with a screw D = 15 mm and L/D 14 and a die head with a diameter of 3 mm and 15 mm length. The extrusion temperature was 155 °C, 185 °C, and 185 °C, respectively, in the first zone, the second zone of the barrel, and the extrusion head. Due to the variable bulk density of the processed material, the rotational speed of the screw was variable and set to obtain an extrusion capacity of 1.2 kg/h. This allowed to maintain a constant residence time of the material in the plasticizing system of the extruder.

The stripped extrudate was cooled in air and then granulated. Finally, PVC composite pellets containing 10 phr, 20 phr, 30 phr, and 40 phr ES (per 100 phr PVC) were produced. These materials are marked PVC/10ES, PVC/20ES, PVC/30ES, and PVC/40ES, respectively. Material free of ES filler, denoted PVC, was produced under the same conditions.

In the next processing stage, the materials were pressed with a hydraulic press at 185 °C. An initial melting time of 3 min and pressing under a pressure of 10 MPa for 2 min were used. Using this method, plates with dimensions of 120 mm × 120 mm × 2 mm, 100 mm × 100 mm × 4 mm, or 100 mm × 100 mm × 1 mm were obtained, from which test pieces were cut out using a CNC milling machine.

### 2.4. Testing Methods of ES and PVC/ES Composites

To characterize the produced filler, sieve analysis, thermogravimetric measurements in nitrogen, SEM observations, and density determination using the psychometric method were performed.

To analyze the influence of the filler on the processing properties, plastographometric measurements were performed and the melt mass flow rate (MFR) was determined. The mechanical properties were assessed by the determination of tensile properties and the Charpy impact strength. In addition, an analysis of the change in the storage modulus and the loss factor was performed using DMA. Thermal properties were determined using the TG thermogravimetric method. Additionally, the thermal and density stability time was determined by the gas pycnometer method. SEM and CT computer microtomography was implemented to investigate the structure of the PVC/ES composites.

#### 2.4.1. SEM Analysis

The sample surface was investigated with a ZEISS AVO 40 scanning electron microscope (SEM) (Carl Zeiss AG, Oberkochen, Germany) after sputter coating with a gold layer. Scanning electron microscopy samples were broken in liquid nitrogen.

#### 2.4.2. Thermogravimetric Analysis (TG)

Thermogravimetric measurements were made with a TG 209 F3 (Netzsch GmbH & Co. Holding KG, Selb, Germany) device in nitrogen. The measurement was carried out in the temperature range of 30–900 °C with a temperature change rate of 10 °C/min. The used sample mass was in the range 10–15 mg, the protective and purge gas flow was 30 mL/min each. From the obtained dependences, the temperature of the loss of 1% (*T*_1_), 5% (*T*_5_), and 50% (*T*_50_) of the sample mass, as well as the temperature of the maximum decomposition rate (*T_DTG_*) and the residue after decomposition at 900 °C (*RM*), were determined. The obtained biofiller and polymer samples were measured in at least two repetitions.

#### 2.4.3. Particle Size Distribution of Filler

The filler’s particle size distribution was characterized using a laser particle sizer Fritsch ANALYSETTE 22 (Idar-Oberstein, Germany) apparatus operating in the range of 0.08–2000 μm.

#### 2.4.4. Plastographometric Analysis

The processing properties were tested using the plastographometric method applying an FDO 234H torque rheometer (Brabender GmbH & Co., Germany). A defined portion (68 g) of the material mixture was poured into the plastographometer chamber with a volume of 50 cm^3^ heated to 185 °C. The torque change and the mass temperature were recorded as a function of the kneading time. The rotor speed was 30 min^−1^. The total processing time of the material in the chamber was 15 min. From the determined plastograms, the following characteristic values were determined: *M_X_*—maximum torque at the fusion point; *T_X_*—the temperature at the fusion point; *t_X_*—time, in minutes, to reach the fusion point; *M_e_* torque at the endpoint; *T_e_*—the temperature at the endpoint [34,44].

#### 2.4.5. Determination of Melt Mass Flow Rate (MFR)

The melt mass flow rate (MFR) was determined by using the standardized (PN-EN ISO 1133) capillary rheometer Dynisco LMI 4001 (Forge Parkway, Franklin, MA, USA). The measurement was carried out at 190 °C with a nominal load of 21.6 kg. The measurement was repeated three times for each type of material. Obtained results were expressed in units of g/10 min.

#### 2.4.6. Density Determination and Evaluation of Porosity

The specific weight of the filler and the obtained composites was determined using the Pycnomatic gas pycnometer by Thermo Fisher Scientific Inc. (Waltham, MA, USA). The measurement was carried out in a helium atmosphere at a pressure of 0.2 MPa, at the flow direction reference first, at 20 °C using a 40 cm^3^ measuring cell. The obtained density test results determined the porosity of composites as the difference between the theoretical and experimental density values. The theoretical density values were calculated according to Equation (1):

*ρ_th_* = *ρ_m_*(1 − *φ*) + *ρ_f_ φ*, (1)

where *ρ_th_*—theoretical density of the composite, g/cm^3^; *ρ_m_*—density of the matrix, g/cm^3^; *ρ_f_*—density of the filler, g/cm^3^; *φ*—a volume fraction of the filler.

The porosity of the material was determined by Equation (2):

*p* = (*ρ_th_* − *ρ_ex_*/*ρ_th_*) 100%, (2)

where *p*—porosity, %; *ρ_ex_*—an experimental density of composite, g/cm^3^ [45].

#### 2.4.7. Time of Thermal Stability (*t_ts_*)

The thermal stability time was determined as the value of the time after which the sample, heated in a constant high temperature, begins to deteriorate, which results in the release of hydrogen chloride, which is evidenced by the color change of the Congo indicator from red to blue. A sample of the material (approx. 0.5 g) was placed in a glass test tube with an internal diameter of 4.7 mm and a wall thickness of 0.65 mm. The Congo red test paper was inserted into the upper part of the test tube to a depth of 3 mm. The tube was placed in a stand so that its end at its three-quarters height was immersed in an oil bath heated to 200 °C [44]. The result of the test is the time, expressed in minutes, for the first visible discoloration of the indicator paper to appear. The results shown are the average of three measurements.

#### 2.4.8. Mechanical Properties

To assess the influence of the biofiller from the hen eggshell waste on the mechanical properties of the PVC/ES composites, tests of the tensile properties and impact strength were carried out.

The tensile mechanical properties were determined in accordance with EN ISO 527. Standardized test specimens (type 5A) were cut with a CNC milling machine from a plate with dimensions of 120 mm × 120 mm × 2 mm. The measurement was carried out on a Zwick/Rolel Z010 testing machine at 23 °C. The test speed was 100 mm/min (for 1 mm/min for a modulus). The modulus of elasticity, maximum stress, and deformation at maximum stress was determined. The measurement was performed on at least five samples for each material.

The impact strength of the obtained composites was determined by the Charpy method following the EN ISO 179-1 standard. For the test, unnotched samples with dimensions of 80 mm × 10 mm × 4 mm were used, cut with a CNC milling machine from a pressed plate with dimensions of 100 mm × 100 mm × 4 mm. A pendulum with a nominal impact energy of 4 J was used. The measurement was performed on at least five samples for each material.

#### 2.4.9. Vicat Softening Temperature (VST)

The Vicat softening temperature (VST) was evaluated following the standard ISO 306:2004, with a heating rate of 50 °C/h and a load of 10 N, with silicon oil used as the heating medium. The VST for each series was determined based on three measurements.

#### 2.4.10. Dynamical Mechanical Thermal Analysis (DMA)

The dynamic mechanical analysis (DMA) tests were carried out using the DMA Artemis Netzsch (Selb, Germany) device operating in the three-point bending system (support spacing 20 mm, sample width 10 mm, thickness 1 mm), with a deformation of 10 μm, in the temperature range of 25–120 °C and a temperature rise rate of 2 °C min^−1^. The distortion frequency was 1 Hz. The relatively low frequency and amplitude of deformations made it possible to perform measurements in the linear range of viscoelasticity and with a low loss modulus [46,47]. The dependence of the storage modulus (*E*′) and the loss factor (tan*δ*) on the temperature was analyzed. Based on the recorded thermograms, *E*′ was determined at 30 °C, 50 °C, and 70 °C. The glass transition temperature range was also determined based on the extrapolated initial (*T_g_* onset), final (*T_g_* offset), and the inflection point (*T_g_* inflection) of the rapid change of the storage modulus curve, and the maximum of the peak of the loss factor curve (tan*δ* peak).

#### 2.4.11. X-ray Microcomputed Tomography (Micro-CT)

Samples cut from the measurement part of molded pieces were investigated by Bruker SkyScan 1173 X-ray microcomputed tomography (Kontich, Belgium) with an image pixel size of 5.16 µm by a source voltage of 50 kV. The NRecon program reconstructed projections, and the analysis was done using the CT Analyzer (CTAn) program, while the CTvox program obtained the 3D visualization of the ES distribution.

#### 2.4.12. Statistical Analysis

Origin 8.6 Pro software with implemented statistical analysis modules was used for statistical analysis of the obtained results. Analysis of variance (ANOVA) with the post hoc Tukey test was used to compare the significant difference for each mean value. The normal distribution was confirmed using the Shapiro−Wilk test, while the Levene test was used for homogeneity of variance. Homogeneous groups within the analyzed properties are indicated by letters in the tables or graphs. All analyses were performed, assuming a significance level below 0.05. The standard deviation is marked as the error bars in the graphs or the results given after mean value for the tabulated values.

## 3. Results and Discussion

### 3.1. Analysis of Eggshell Filler (ES)

The filler in the form of crushed silica eggs, produced in accordance with the research methodology, was characterized in terms of its application for the production of the PVC/ES composites. The particle size distribution as well as the shape and dimensions of the filler were assessed. Thermogravimetric analysis was performed to determine the thermal stability of the filler.

#### 3.1.1. SEM Observation of ES Filler

**Figure 1:**
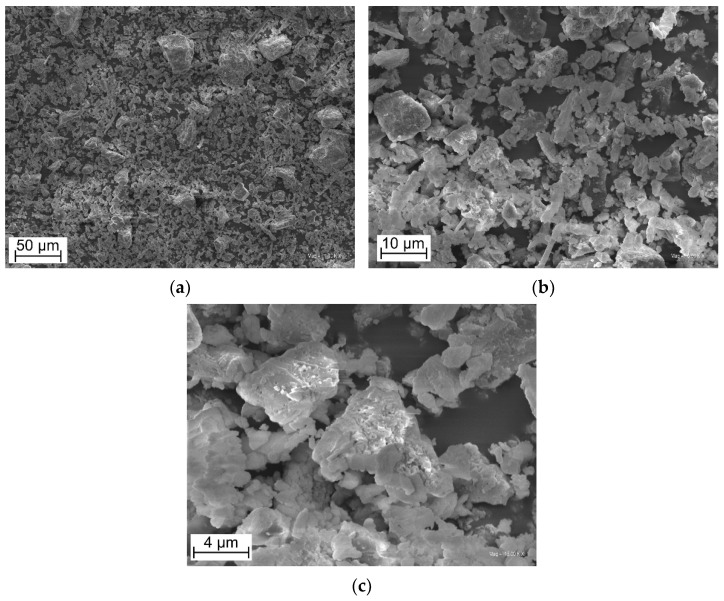
SEM micrographs of obtained ES powder (magnification (**a**) −1 k×, (**b**) −5 k×, (**c**) 15 k×).

Figure 1 presents SEM micrographs of the obtained ES filler. The presence of an irregularly shaped particle was observed for the ES powder. The filler particles are irregular in shape and have sharp edges. The smaller particles are in the lamellar form, while the larger ones are in the “lumpy” form. There are also visible pores in the surface structure of the filler, occurring naturally in the eggshell. This spongy, porous crust-building calcium carbonate structure is partially retained in the filler. The filler particles form clusters caused by the strong bonds between the natural proteins with calcium ions (Ca^2+^) [48]. According to the literature, the BET surface area was predicted at 8.4522 m^2^/g. The pore volume and pore size were found to be 0.046 cm^3^/g and 255.143 Å, confirming that the eggshell is a macroporous material with a spongy structure [49].

#### 3.1.2. Thermogravimetric Analysis (TG)

**Figure 2:**
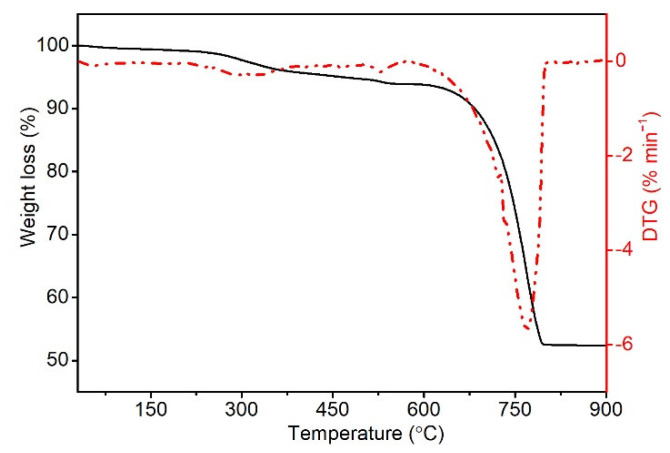
TG thermogram of ES filler in nitrogen.

Figure 2 shows the TG thermogram of the produced ES filler in nitrogen. In the temperature range of 70–85 °C, a slight loss of mass of the sample related to water evaporation was observed [28]. The decomposition in the range of 260–340 °C, amounting to approx. 5.9%, is assigned to the decomposition of the organic substance contained in the ES. Higher decomposition temperatures of organic parts of the shell are associated with their strong interaction with calcium carbonate, which remained attached to the shell despite their initial washing away [49,50]. Decomposition in the range of 650–770 °C, 36.5%, is related to the decomposition of calcium carbonate concomitant with the evaporation of carbon dioxide and formulation of calcium oxide. The residue mass after decomposition at 900 °C is 49.3%. The proportion of organic parts in the obtained filler is about 6%, and the mineral part is about 93%, which is very consistent with previous literature reports [28]. The obtained biofiller, in the PVC processing temperature range, i.e., up to 200 °C, shows a slight loss of sample mass (1.5%), which may be related to the decomposition of the organic residue not removed during the washing of the shells.

#### 3.1.3. Particle Size Distribution

**Figure 3:**
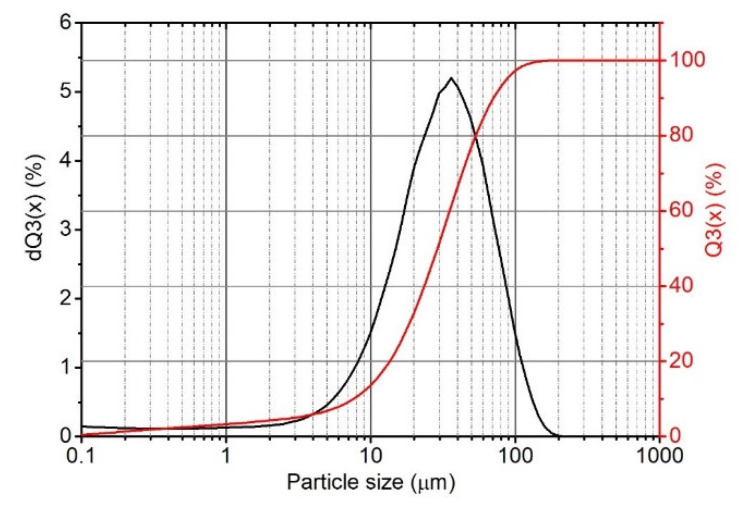
Particle size distribution of eggshell filler.

Based on the cumulative curve (Figure 3), the average particle size of the filler (D50%) was determined to be 29.0 µm, with particles with a diameter of 40.5 µm being the particles with the highest frequency of occurrence. Additionally, 10% of the particles are smaller than 7.6 µm (D10%) and larger than 70.7 µm (D90%).

### 3.2. Properties Analysis of PVC/ES Composites

PVC/ES composites have been successfully produced. For the obtained composites, an analysis of both processing and functional properties was performed. The influence of the filler on the processing properties was carried out on the basis of plastographometric and MFR tests. The thermal stability time and the decomposition temperature of the PVC, important for processing reasons, were also performed. Mechanical and thermomecanical tests were assessed on the basis of tensile properties, VST, DMA, and impact strength. The analysis of composites was supplemented with structural studies using SEM and computer microtomography.

#### 3.2.1. Plastographometric and MFR Analysis

**Figure 4:**
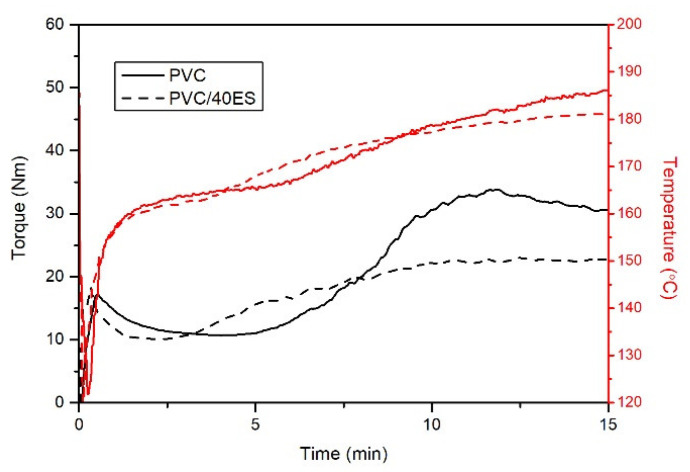
An Exemplary plastogram of PVC and PVC/40ES processing.

Figure 4 shows exemplary processing plastograms determined for PVC and PVC containing 40 phr ES filler. The characteristic values read out, following the research methodology, are summarized in Table 1.

Adding fillers to PVC very often shortens the fusion time, accompanied by a rapid increase in the torque [44,51,52,53]. In the case of using ground eggshells, different relationships were observed. It was found that the fusion time *t_x_* was shortened only slightly for composites with 10 phr of filler. At the same time, there was no difference in the *T_X_* temperature value and no significant reduction in the torque value at the fusion point (*M*_X_). Notably, the addition of ES does not increase the torque at the end of the test *M_e_* concerning PVC; on the contrary, it reduces it. It is also related to the lower temperature *T*_e_, which is related to the lack of self-heating of the material as a result of energy dissipation during processing. The *M_X_* and *M_e_* torque reduction may result from the higher density of PVC/ES composites, which is related to the lower degree of filling of the plastographometer chamber [54]. At the same time, it should be noted that the addition of a filler to polymers increases their viscosity. In the case of PVC/ES composites, the proportion of the filler does not change the viscosity of the processed composite to such an extent as to increase the recorded torque value. It should be noted that the *M_e_* values characterizing PVC/ES composites are determined for the lower temperature of the processed composite, which an increase in viscosity should accompany. The described relationships may result from the significant fragmentation of eggshells, which is not conducive to the initial grinding of PVC grains and show an additional lubricating effect observed for other polymers/filler systems [34,55]. The composition of the PVC blend is also essential here, as it contains process additives to facilitate the processing of highly filled composites. ES does not deactivate the action of these agents, which in turn confirms that generally available process additives can be used to produce the composition of dry blends dedicated to the production of PVC/ES. Summarizing the plastographometric tests, it can be stated that the filler in the form of crushed chicken eggshell has a positive effect on the processing properties of the PVC without causing significant changes in the fusion time and limiting the self-heating effect of the material, which may significantly affect the phenomena of PVC degradation during processing.

MFR plays a curial role in controlling the processability of thermoplastic materials. The introduction of fillers to polymeric materials significantly increases the viscosity of the plasticized composite, which is manifested by a significant reduction in the value of the mass melt flow rate. The determined values of the MFR for the tested materials are summarized in Table 1. The introduction of ES into the PVC matrix reduces the MFR value from 9.56 g/10 min to about 6 g/10 min. Importantly, no significant increase in the viscosity of the PVC/ES composite was found in the tested range of the ES share. Undoubtedly, the obtained results are influenced by the applied system of modifiers of the PVC processing properties, which facilitates the melt flow of composite [34], the good homogeneity of the produced composites, and the good fragmentation of the ES [56,57]. These results confirm the observations and conclusions drawn during the plastographometric examinations.

#### 3.2.2. Density and Porosity

Table 2 summarizes the results of the density tests of the produced composites, PVC, ES, and the calculated values of the volume fraction of the filler and porosity.

The density of the obtained filler was 2.27 g/cm^3^, which is definitely higher than the PVC. This affects the gradual increase in the density of the PVC composites. The PVC/40ES density was 1.61 g/cm^3^, a 19% increase in density concerning the PVC. Increasing the composite density is not beneficial. However, it is an obvious and expected effect in producing most polymer composites with mineral fillers.

The analyzed materials are characterized by low porosity, which excludes the emission of significant amounts of gases during processing, and a strong influence of this parameter on the mechanical properties of the PVC/ES composites [58,59].

#### 3.2.3. Thermogravimetric Analysis (TG)

**Figure 5:**
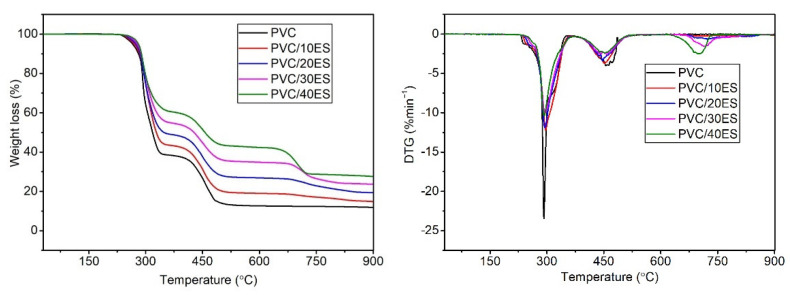
TG thermograms of PVC and PVC/ES composites in nitrogen.

Figure 5 present TG thermograms of the PVC and PVC/ES composites, and Table 3 shows the analyzed values of the characteristic thermal properties.

The decomposition of the PVC in a nitrogen atmosphere takes place in two stages. The first step in the temperature range of 230–350 °C is associated with the dechlorination of the PVC macromolecules and the creation of conjugated polyene structures. The second step in the temperature range of 350–500 °C relates to another further thermal decomposition of the carbonaceous conjugated polyene sequences following the formation of residual chars [36,60,61]. When analyzing the TG thermograms of the PVC/ES composites, an additional stage of weight loss in the range of 650–770 °C related to biofiller presence was observed. As shown in its TG analysis, this stage corresponds to the decomposition of calcium carbonate and the release of CO_2_. As the filler content in the matrix increases, the rate of PVC degradation associated with dehydrochlorination (the first peak on the DTG curve) decreases, as evidenced by the decrease in the maximum height of the DTG peak.

The addition of ES improves the thermal stability of composites, as evidenced by the increase in the decomposition temperature of *T*_1_, *T*_5_, and *T*_50_, along with the increase in the proportion of filler in the PVC. The introduction of 40 phr of the filler increases the temperature of initial decomposition defined as *T*_1_ and *T*_5_ by approx. 16 °C and 15 °C in comparison to the unmodified PVC matrix.

Filler particles with a highly porous structure are able to sorb Cl radicals which are formed during the heating of the PVC, and finally influence the increase of thermal stability of the material. Thereby, the Cl radical acceleration effect on the PVC macromolecules decomposition is limited. Similar effects were observed in the case of the use of calcium carbonate or dolomite [62,63]. Murugan S. attributed the improved thermal stability of the soft PVC as an effect of the thermal insulation of filler. Moreover, he found that larger particle bio-based fillers provide better thermal stability of the composites [38].

#### 3.2.4. Time of Thermal Stability (*t_ts_*)

**Figure 6:**
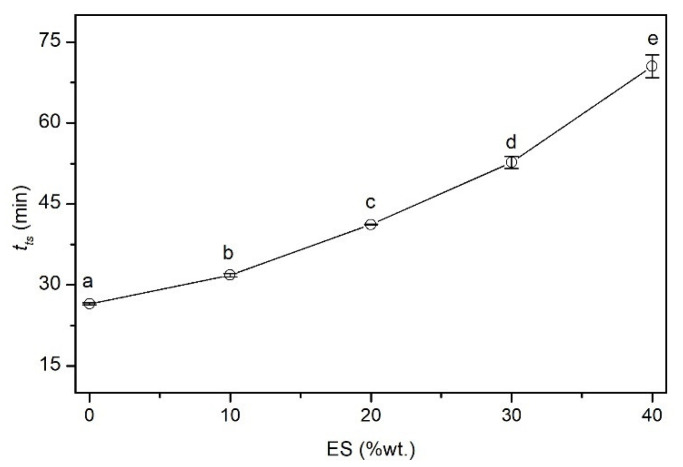
Time of thermal stability (*t_ts_*) as a function of the biofiller content in the PVC composite.

Figure 6 shows the dependence of the changes in the thermal stability time (*t_ts_*) as a function of the biofiller content in the PVC composite. The time of thermal stability, i.e., the time after which the destruction of the poly(vinyl chloride) heated at 200 °C begins, which is manifested by the release of a gaseous HCl as a decomposition product, was 26.5 min for the unfilled PVC. Introducing a biofiller into the matrix extends the time of the thermal resistance of the composites. Adding a small amount of ES (10 phr) causes a significant elongation of *t_ts_* (about 20%) compared to the unfilled PVC. Increasing the proportion of the ES to 40 phr increases the stability time by as much as 166%. These results indicate the beneficial effect of ground hen eggs on extending the thermal stability of the PVC composites following the mechanism described in the TG analysis and the higher specific surface area of the filler in contact with the heated PVC macromolecules. The protective influence against the heating of the PVC is also possible [64,65]. However, the explanation for extending the thermal stability time by as much as 44 min due to changing only the thermal conductivity value does not seem justified. It is also possible that the favorable influence of the ES on the processing properties, as shown based on plastographometric tests, decreased the temperature of the composite during the extrusion process. This may additionally affect the elongation of the *t_ts_* of the produced materials with a biofiller.

Undoubtedly, the reaction of the PVC dechlorination with CaCO_3_ presented in [66,67] follows the simplified reaction:

PVC + CaCO_3_ → [-CH = CH-] + CO_2_ + H_2_O + CaCl_2_, (3)

has an impact on the improvement of the thermal stability of the PVC.

On this basis, it can be concluded that the addition of ES may, in the initial stage of PVC destruction, especially mechanothermally induced, protect the macromolecules against the catalytic effect of the formed Cl radicals. This mechanism involves catching and binding Cl free radicals to the form of CaCl_2_, thus extending the time of the PVC thermal stability. ES acts as a radical scavenger. An additional factor here is the significant preserved natural porosity and specific surface of the filler, which is used primarily in catalytic applications [42]. Increasing PVC thermal stability by adding mineral fillers containing calcium carbonate is not apparent. Slight thermal stability deterioration was found due to the addition of chalk. Additionally, insufficient filler dispersion in the matrix and its agglomeration may contribute to the deterioration of thermal stability [68].

#### 3.2.5. Mechanical Properties

The results of tests of mechanical properties of PVC and PVC/ES composites are summarized in Table 4.

By analyzing the obtained impact strength results, it can be concluded that the PVC composites containing up to 30 phr of filler do not show a significant difference in impact strength compared to the PVC. However, each successive increase in the content of the filler significantly increases the value of the material’s modulus of elasticity. This indicates the same adhesion between the polymer matrix and the filler. However, the symptoms of reinforcement are manifested only in the elastic regime. In the plastic regime, at higher deformations (the modulus was determined up to 0.25% deformation), the reinforcement effect is not observed. A decrease in the tensile strength and a reduction in the value of the elongation at break have been found. The increase in the elongation at the break between the PVC and the composite containing 10 phr of the filler is related to the change of the fusion degree and the increased grinding of the PVC micrograins [69,70] rather than the active action of the filler. In this case, the beneficial effect of the ES on the processing properties of the PVC is again visible.

It should be added that, in the case of the filler used, its surface modification was not carried out, which is common in the case of conventional calcium carbonate used for PVC modification. Such an adopted research path enables the assessment of PVC/ES interactions. However, in further research, it will be beneficial to use the surface modification of the filler to use its ES potential more effectively. Nevertheless, the mechanical and processing properties of the obtained composites do not disqualify them from being used as a valuable material for producing, e.g., thick-walled profiles or injection details.

#### 3.2.6. Vicat Softening Temperature (VST)

**Figure 7:**
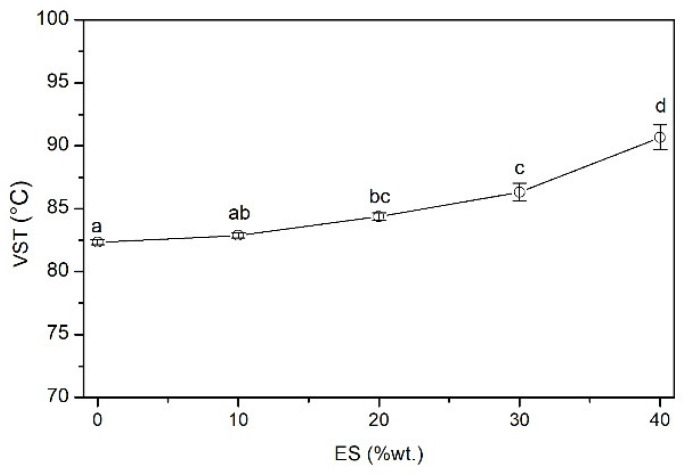
Vicat softening temperature (VST) vs. ES content.

Figure 7 shows the influence of the ES content on the VST value of the PVC/ES composites. Along with the increase in the concentration of the filler, a significant increase in the discussed value is observed. The softening temperature of the PVC/40ES composite is 10% higher than that of the PVC. These observations are most likely the result of the increased interactions between the polymer matrix and the filler, which were already observed during the analysis of the mechanical properties. The increase in the VST value proves that these interactions are also maintained at higher temperatures.

#### 3.2.7. Dynamical Mechanical Thermal Analysis (DMA)

**Figure 8:**
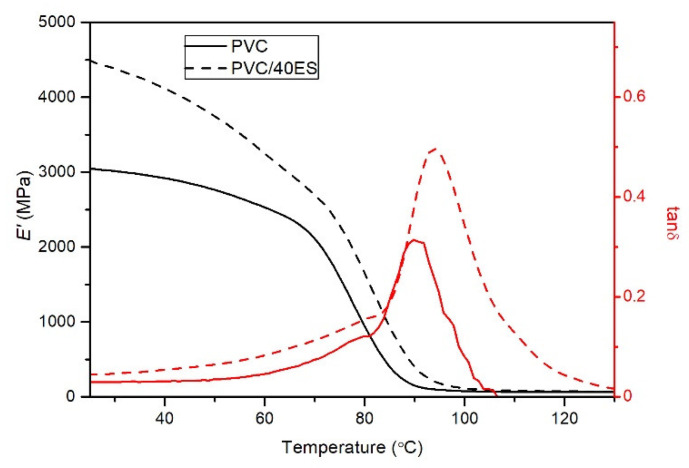
Thermograms of DMA analysis of PVC and PVC/40ES.

Figure 8 shows the example of storage modulus (*E*′) and the loss factor (tanδ) as a function of temperature determined for the PVC and the PVC/40ES composite. Characteristic values are presented in Table 5.

The DMA analysis confirms the conclusions stated from the mechanical properties and the softening temperature analysis. As in the case of the mechanical tensile properties, a significant and clear increase in the material’s stiffness was found with the increase in the proportion of the filler. In addition, based on the analysis of the change in the *E***′** value from temperature, it was found that the stiffening effect of ES is also observed in the range of higher temperatures, up to the glass transition temperature (values determined at 70 °C).

**Figure 9:**
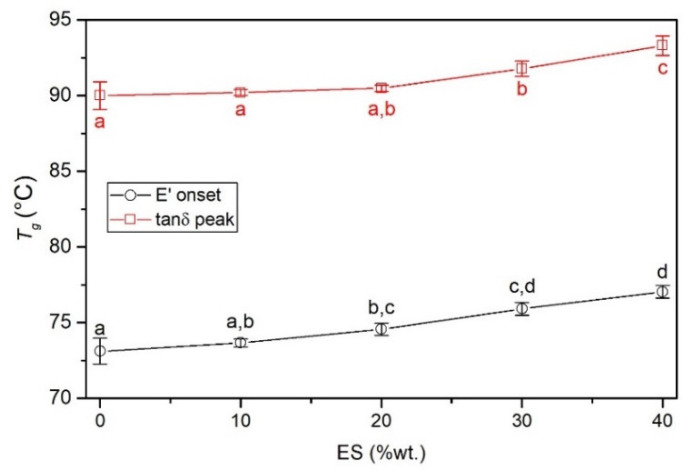
Glass transition (*T_g_*) vs. content of ES filler.

Due to the different methodology for determining the glass transition temperature from DMA thermograms, an analysis of the entire glass transition range is presented. The most significant differences between the determined values of *T_g_* for the same material are characterized by the values established at the beginning of the change *E*′ and the maximum tan*δ*. These values are additionally summarized in Figure 9.

The increase of the ES content in the PVC results in a slight but significant increase in the glass transition temperature. This difference is from 3.3 °C to 3.9 °C, depending on the determination methodology. Although the *T_g_* value increases slightly, this proves the interaction between the ES filler and the poly(vinyl chloride). The ES particles located in the polymer matrix significantly hinder the movement of segments of macromolecules responsible for the occurrence of the highly elastic state.

#### 3.2.8. SEM Analysis

**Figure 10:**
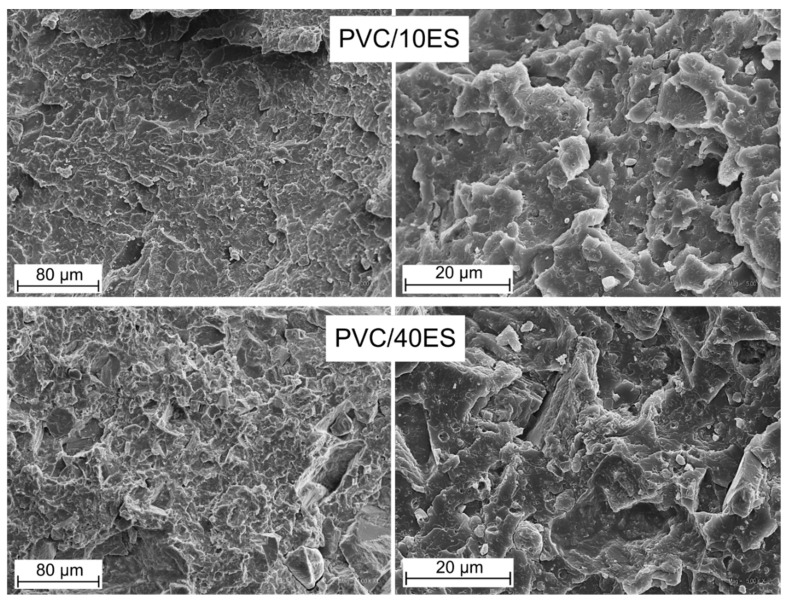
SEM images of the cryogenic breakthroughs of composites containing 10 phr and 40 phr filler of 1 k× and 5 k×.

The SEM images (Figure 10) show the cryogenic breakthroughs of composites containing 10 phr and 40 phr filler at a magnification of 1 k× and 5 k×. A fine dispersion of the biofiller in the PVC matrix was observed. Larger fragments of the ES particles are also observed among smaller, firmly embedded particles in the PVC matrix of bionic chalky filler with a size of up to 10 μm. With large filler particles, there is a clear filler/matrix boundary in the form of caverns and cracks in the structure. This effect may, at a higher concentration of such particle size, constitute weak points of mechanical stress transfer and thus weaken the mechanical properties of the material. A way to counteract such effects may be the use of ES with a high degree of fragmentation in the future.

Additionally, it would be advantageous in the future to carry out surface modifications to increase the compatibility of the matrix and the filler, as in the case of conventionally used calcium carbonate modifications. It was also observed that the number of discontinuities and defects in the structure increased with the amount of filler introduced into the matrix. It is also confirmed by the calculated porosity of composites, which reaches the maximum values with the highest share of the ES in the PVC matrix.

#### 3.2.9. Micro-CT

**Figure 11:**
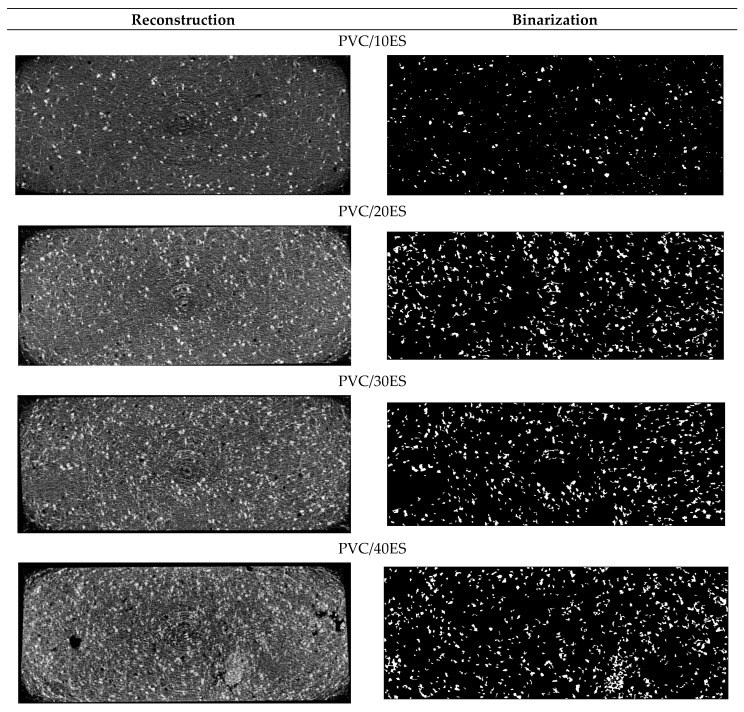
Single layers/cross-sections after reconstruction (**left column**) and the same layers after binarization (**right column**), with white spots as ES particles.

The advantage of X-ray microcomputed tomography is enabling the nondestructive investigation of the material in its whole volume [71]. Figure 11 shows the reconstructed single layers of sample cross-sections, which are in size close to the dimensions of the investigated pieces, which are 4 mm × 10 mm. The rings in the picture center are so-called ring artifacts. The right column of Figure 11 presents single layers after binarization, with white spots as ES particles. Micro-CT confirms increasing amounts of voids with ES particle amounts.

**Figure 12:**
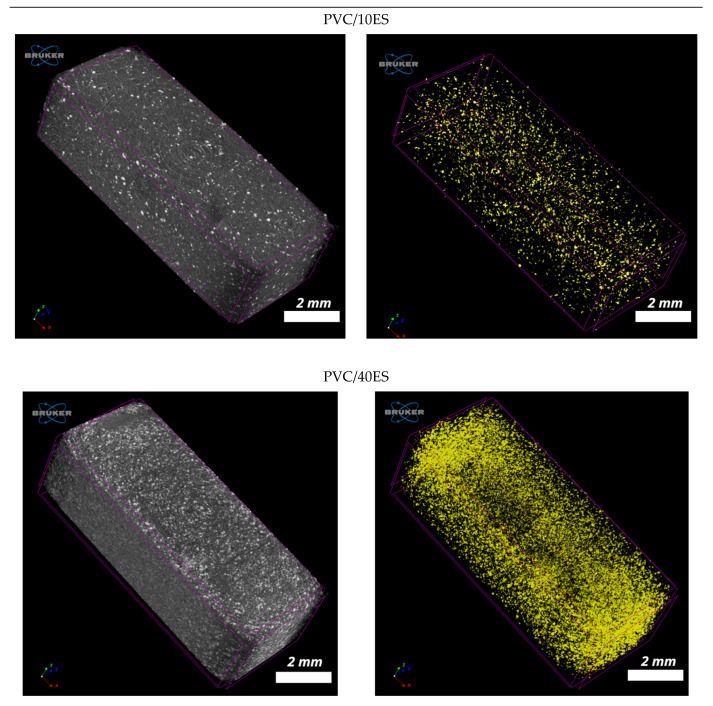
The 3D visualizations of pieces scanned with microcomputed tomography (the **left column**) and the same pieces after removing the PVC matrix, with ES particles as yellow spots.

The average values of the ES particle sizes obtained with 3D analysis by the CTAn program are presented in Table 6. The average values are slightly lower when comparing the results obtained with a laser particle sizer but comparable to the 40.5 µm diameter mostly frequent fraction. It is probably an effect of the decreasing particle size during the polymer processing. The 3D visualizations of the scanned pieces by the CTvox program and the ES particle distribution in a PVC matrix are presented in Figure 12.

## 4. Summary

Composites of unplasticized poly(vinyl chloride) with milled eggshell waste were produced successfully.

It was found that the addition of ES has a beneficial effect on processing properties, particularly during PVC fusion. The filler does not significantly change the fusion time and reduces the self-heating impact of the material, which may dramatically affect the phenomena of PVC degradation during processing. In addition, generally available process additives can be used in the composition of dry blends dedicated to producing PVC/ES.

The beneficial effect of ES filers on improving the thermal stability of PVC has been demonstrated. The addition of ES significantly increased the thermal stability temperature and the thermal stability time. ES most likely acts as a Cl radical scavenger in the initial stage of PVC destruction.

A significant increase in the modulus of elasticity was found along with the increase of the ES concentration, with a simultaneous decrease in the tensile strength. Nevertheless, the mechanical properties of the obtained composites do not disqualify them from being used as a valuable material for producing, e.g., thick-walled profiles or injection details.

ES particles located in the PVC matrix significantly hinder the movement of segments of macromolecules, as evidenced by a significant increase in the VST and a significant increase in the glass transition temperature.

The filler in the form of crushed chicken eggshells is valuable for PVC modification. Its use is a good form of waste management. Moreover, the characterization of PVC/ES composites encourages further application studies, particularly in surface modifications aimed at increasing the compatibility of the matrix and the filler, as in the case of conventionally used calcium carbonate modifications.

Furthermore, the incorporation of ES in polymeric materials will promote ecofriendly material construction. In addition, in a combination with PVC, such materials after use exhibit, according to the literature, a more environmentally friendly and efficient method of disposal, which may contribute to increasing the recyclability of PVC.

Hen eggshells could become a fully exploitable raw material for polymer modification and many other applications, assuming the organization of selective collection of this waste from industrial egg utilization sites, i.e., food factories, poultry farms, and bakeries, which would ensure the increased access to this waste.

## Figures and Tables

**Table 1:** Values of plastografometric and MFR analysis.

Sample	*t_X_* (min)	*M_X_* (Nm)	*T_X_* (°C)	*M_e_* (Nm)	*T_e_* (°C)	*MFR* (g/10 min)
PVC	11.8 ± 0.15 ^b^	33.9 ± 0.20 ^a^	181.9 ± 0.72 ^a^	30.6 ± 0.47 ^a^	186.2 ± 0.21 ^a^	9.56 ± 0.25 ^a^
PVC/10ES	10.0 ± 1.02 ^a^	29.5 ± 0.44 ^b^	179.9 ± 0.51 ^a^	25.7 ± 0.31 ^b^	183.5 ± 0.49 ^b^	6.39 ± 0.12 ^b^
PVC/20ES	11.4 ± 0.58 ^a,b^	25.3 ± 0.49 ^c^	180.8 ± 0.49 ^a^	24.6 ± 0.25 ^c^	182.1 ± 0.12 ^c^	6.28 ± 0.25 ^b^
PVC/30ES	12.1 ± 0.30 ^b^	24.4 ± 0.20 ^d^	181.0 ± 0.95 ^a^	23.7 ± 0.10 ^d^	181.9 ± 0.15 ^c^	5.70 ± 0.41 ^c^
PVC/40ES	12.3 ± 0.31 ^b^	23.0 ± 0.13 ^e^	180.1 ± 1.0 ^a^	22.7 ± 0.21 ^e^	181.0 ± 0.30 ^d^	5.88 ± 0.21 ^b,c^

**Table 2:** Results of density and porosity calculation for PVC, ES, and PVC/ES composites.

Sample	Density (g/cm^3^)	Volume Fraction ES	Porosity (%)
EGS	2.27 ± 0.002	-	
PVC	1.35 ± 0.012	-	
PVC/10ES	1.40 ± 0.001	0.063	0.661
PVC/20ES	1.47 ± 0.001	0.135	0.451
PVC/30ES	1.54 ± 0.001	0.216	0.854
PVC/40ES	1.61 ± 0.002	0.308	1.804

**Table 3:** Results of TG analysis.

Sample	*T*_1_ (°C)	*T*_5_ (°C)	*T*_50_ (°C)	*T_DTG_* (°C)	*RM* (%)
PVC	240.4	265.2	318.5	291.4	11.9
PVC/10ES	244.9	269.8	328.1	296.4	14.8
PVC/20ES	249.1	273.9	347.9	294.7	19.3
PVC/30ES	253.3	277.5	427.3	295.3	23.6
PVC/40ES	256.7	280.8	456.2	292.7	27.6

**Table 4:** Mechanical properties of PVC and PVC/ES composites.

Sample	*E_t_* (MPa)	*σ_M_* (MPa)	*ε_b_* (%)	*a_cU_* (kJ/m^2^)
PVC	1235 ± 17.6 ^a^	46.5 ± 1.26 ^a^	5.9 ± 0.54 ^c^	17.4 ± 1.17 ^a^
PVC/10ES	1380 ± 18.9 ^b^	42.6 ± 0.57 ^b^	20.9 ± 2.03 ^a^	16.2 ± 2.26 ^a^
PVC/20ES	1532 ± 20.5 ^c^	35.3 ± 0.85 ^c^	11.2 ± 1.47 ^b^	18.2 ± 2.87 ^a^
PVC/30ES	1698 ± 19.4 ^d^	29.6 ± 0.78 ^d^	5.8 ± 1.34 ^c^	15.1 ± 1.88 ^a^
PVC/40ES	1787 ± 45.7 ^e^	21.2 ± 0.84 ^e^	4.1 ± 0.58 ^c^	10.1 ± 1.51 ^b^

**Table 5:** Characteristic values of DMA analysis.

Sample	*T_g_* (°C)				*E*′ (MPa)		
	*E*′ Onset	*E*′ Inflection	*E*′ Offset	tan*δ* Peak	30 °C	50 °C	70 °C
PVC	73.1 ± 0.86 ^a^	78.4 ± 0.58 ^a^	83.8 ± 0.64 ^a^	90.0 ± 0.92 ^a^	2946 ± 80.2 ^a^	2720 ± 74.2 ^a^	2206 ± 29.7 ^a^
PVC/10ES	73.6 ± 0.27 ^a,b^	78.7 ± 0.12 ^a,b^	84.2 ± 0.20 ^a,b^	90.2 ± 0.20 ^a^	3070 ± 106.9 ^a^	2809 ± 107.8 ^a^	2346 ± 29.0 ^a^
PVC/20ES	74.6 ± 0.39 ^b,c^	79.6 ± 0.19 ^b^	84.9 ± 0.16 ^b^	90.5 ± 0.21 ^a,b^	3593 ± 83.5 ^b^	3259 ± 78.6 ^b^	2565 ± 18.4 ^b^
PVC/30ES	75.9 ± 0.42 ^c,d^	80.8 ± 0.29 ^c^	86.3 ± 0.30 ^c^	91.8 ± 0.50 ^b^	4003 ± 97.4 ^c^	3606 ± 75.4 ^c^	2795 ± 62.2 ^c^
PVC/40ES	77.0 ± 0.42 ^d^	81.8 ± 0.26 ^d^	87.5 ± 0.34 ^d^	93.3 ± 0.65 ^c^	4356 ± 99.9 ^d^	3865 ± 97.5 ^d^	2782 ± 68.9 ^c^

**Table 6:** Average ES particle size obtained with 3D analysis by the CTAn program.

Sample	Average Particle Size (µm)
PVC/10ES	37.39
PVC/20ES	35.00
PVC/30ES	38.10
PVC/40ES	34.91

## Data Availability

The data supporting this study’s findings are available from the corresponding authors (Katarzyna Skórczewska, Krzysztof Lewandowski, and Piotr Szewczykowski) on request.
